# Bilateral actigraphic quantification of upper extremity movement in hemiparetic children with perinatal stroke: a case control study

**DOI:** 10.1186/s12984-021-00962-9

**Published:** 2021-12-16

**Authors:** Asha Hollis, Lauran Cole, Ephrem Zewdie, Megan J. Metzler, Adam Kirton

**Affiliations:** 1 Pediatrics, Cumming School of Medicine, University of Calgary, Calgary, Canada; 2 Neurosciences, Cumming School of Medicine, University of Calgary, Calgary, Canada; 3 Medicine, Cumming School of Medicine, University of Calgary, Calgary, Canada; 4 Clinical Neurosciences, Alberta Children’s Hospital, Calgary, Canada; 5 Pediatric Neurology, Alberta Children’s Hospital, 28 Oki Drive NW, Calgary, AB T3B6A8 Canada

**Keywords:** Hemiparetic cerebral palsy, Bilateral actigraphy, Real-life activity, Upper-extremity movement, Pediatrics

## Abstract

**Background:**

Hemiparetic cerebral palsy impacts millions of people worldwide. Assessment of bilateral motor function in real life remains a major challenge. We evaluated quantification of upper extremity movement in hemiparetic children using bilateral actigraphy. We hypothesized that movement asymmetry correlates with standard motor outcome measures.

**Methods:**

Hemiparetic and control participants wore bilateral wrist Actiwatch2 (Philips) for 48 h with movement counts recorded in 15-s intervals. The primary outcome was a novel statistic of movement asymmetry, the Actigraphic Movement Asymmetry Index (AMAI). Relationships between AMAI and standard motor outcomes (Assisting Hand Assessment, Melbourne Assessment, and Box and Block Test [BB]) were explored with Pearson or Spearman correlation.

**Results:**

30 stroke (mean 11 years 2 months (3 years 10 months); 13 female, 17 male) and 23 control (mean 11 years 1 month (4 years 5 months); 8 female, 15 male) were enrolled. Stroke participants demonstrated higher asymmetry. Correlations between AMAI and standard tests were moderate and strongest during sleep (BB: *r* = 0.68, *p* < 0.01).

**Conclusions:**

Standard tests may not reflect the extent of movement asymmetry during daily life in hemiparetic children. Bilateral actigraphy may be a valuable complementary tool for measuring arm movement, potentially enabling improved evaluation of therapies with a focus on child participation.

**Supplementary Information:**

The online version contains supplementary material available at 10.1186/s12984-021-00962-9.

## Background

Cerebral palsy (CP) accounts for most lifelong neurological disability and affects more than 17 million people worldwide [1–4]. Patients with hemiparetic cerebral palsy (HCP) suffer from motor dysfunction on one side of the body, often a result of acquired injury to the motor system, including the motor cortex or corticospinal tracts [1, 5]. The leading cause of HCP is perinatal stroke due to a focal disruption in cerebral blood flow between 20 weeks gestation and 28 days postpartum [1, 6, 7]. As a focal injury of defined timing in an otherwise healthy brain, perinatal stroke represents an ideal human model of developmental plasticity [8]. Since HCP is highly prevalent and treatment options are limited, new therapies are required to improve motor outcomes. Developmental preclinical and human models have facilitated clinical trials of non-invasive neuromodulation in hemiparetic children [9, 10]. However, these and other promising trials could benefit from tools capable of measuring bilateral movement during real-life activities.

Validated tools to assess motor function in HCP are established. One example is the Assisting Hand Assessment (AHA), a bilateral measure of performance in which the affected arm may “assist” the unaffected arm in a series of predefined functional tasks [11]. As the AHA is designed to elicit bimanual interaction with objects, it may ultimately overestimate affected arm movement in day-to-day activities [12]. Another example is the Melbourne Assessment (MA), a unilateral upper extremity functional measure in which omission of bimanual function limits generalization to daily activity [13–15]. The Box and Block Test (BB) measures unilateral grasp and reach but offers limited insight into day-to-day function [11, 16, 17]. Although test–retest and inter-rater reliability are established, the AHA and MA are resource-intensive [16].

Such clinical measures are important for health providers to determine what functions and/or skills to target in therapy and to evaluate the success of therapy. However, they do not provide a full picture of function. Currently available measures suffer from limited ability to quantify upper extremity use in affected individuals in their natural environment. Aslam et al. point out that these standardized tests are validated, but reflect specific domains of function within the International Classification of Functioning, Disability, and Health (ICF) [18]. The ICF framework for functioning includes the domains of body structures, body functions, environment, and activity limitation in addition to highlighting interactions between domains. Standard clinical tests such as the AHA evaluate activity well, but do not provide an understanding of how activity limitations play out in an individual’s real-life environment. In-situ assessment of unilateral and bilateral movement is potentially critical to a more complete picture of function for children with hemiparesis. One promising tool which may be able to address this need is actigraphy, a wearable technology. Actigraphs are lightweight, wrist-worn accelerometers that capture objective, detailed movement data in real time during normal community-based activities. When the watches accelerate, a voltage is produced which corresponds to the degree of acceleration, enabling actigraphy to reflect various intensities in movement [7, 19]. Additional advantages include simplicity of use, and no lower age limit for application [15, 19–21]. As with other tools, actigraphy has challenges, including expensive devices, potential for missing data from participant noncompliance, and a lack of established guidelines for data analysis.

Actigraphy has seen limited use in children with HCP. Evidence supports the utility of actigraphic measures of motor function in normal children [19], adults with stroke [7], and some CP populations [20, 22–24], and suggests that bilateral actigraphy can measure real-world motor asymmetry in hemiparetic subjects [24–28]. Beani et al. collected bilateral actigraphy for typically-developing and hemiparetic children while they completed the AHA. Two main findings were reported: (1) they confirmed the validity of bilateral ActiGraph GT3X to measure upper limb motor asymmetry between typically-developing controls and children and youth with hemiparetic cerebral palsy; and (2) they demonstrated that an asymmetric activity count index (from actigraphic data) corresponds to impairment level in hemiparetic children [23]. Similarly, Hoyt et al. showed that accelerometers can detect asymmetries in upper extremity movements [28]. In further studies, they found moderate correlations between accelerometry metrics and the MA, demonstrating construct validity [24]. Actigraphy can only estimate movements and is therefore not a measure of function. However, in a child with hemiparesis, an ability to detect and quantify a relative change in movement of the impaired limb may be a useful proxy of spontaneous use and participation in normal life. Actigraphy thus represents a potentially valuable opportunity to better understand real-life outcomes in children with disabilities.

We aimed to evaluate the efficacy of bilateral actigraphy to quantify upper extremity movement in children with perinatal stroke and HCP, hypothesizing that an actigraphic movement asymmetry index (AMAI) would positively correlate with standard clinical measures of upper extremity function. We also performed an exploratory analysis of the relationship between these standard measures and actigraphic data in relation to different levels of activity intensity.

## Methods

### Populations

Following approval by the Research Ethics Board of the University of Calgary (REB15-1742), two populations of participants were recruited for a case–control study from June 2016 to February 2017. Children with stroke aged 1 to 18 years with Magnetic Resonance Imaging-confirmed ischemic perinatal stroke (arterial or venous) were identified from a population-based research cohort (Alberta Perinatal Stroke Project) [29]. Additional criteria included unilateral stroke and no additional neurological conditions, severe developmental delays, or unstable epilepsy.

Typically developing participants aged 1 to 18 years were recruited from the Healthy Infants and Children Clinical Research Program (HICCUP, www.hiccupkids.ca). Typically developing participants had no neurological conditions or medications. Selection bias was minimized by recruiting comparable ages between stroke and control groups. All participants provided written informed consent/assent.

### Actigraphy

Following recruitment, participants were fitted with actiwatches (Actiwatch2, Philips Respironics, Pennsylvania) on both wrists at the Alberta Children’s Hospital. Watches recorded movement data for a period of 48 h during routine life. Participants (or parents) were asked to record sleep and wake times on a standardized diary and press the event marker button at bedtime and wake-time [30]. Motion data was captured in 15-s epochs. Actiware software (Philips Respironics) generated an Activity Count for each epoch. Times were manually excluded if the participant reported in the diary removal of the watches, such as bathing, as the watches were not waterproof. Rest intervals were generated from participants pressing the watch marker button at bedtime and wake-time and were validated using reported diary sleep and wake times. Sleep intervals were automatically determined by the Actiware sleep interval detection algorithm and could only occur within a rest interval.

Data was segmented according to intervals and/or activity levels. Intervals were segmented into [a] active, [b] rest, [c] sleep, and [d] all. Activity levels were defined for each 15-s epoch by the sum of the Activity Counts for both hands: [a] very low (total of 0–30), [b] low (31–160), [c] moderate (161–524), [d] high (525–812), [e] very high (813+), and [f] all. Levels chosen matched those previously described [19] with the exception of two changes: an additional range of “very low” was added to expand analysis of low level movement, and all range limits were doubled to account for bimanual movement. Segmentation by both interval and activity level separated the data into 24 subgroups. It was therefore possible to have activity of different levels during all intervals including during sleep.

### Statistics

**Fig. 1:**
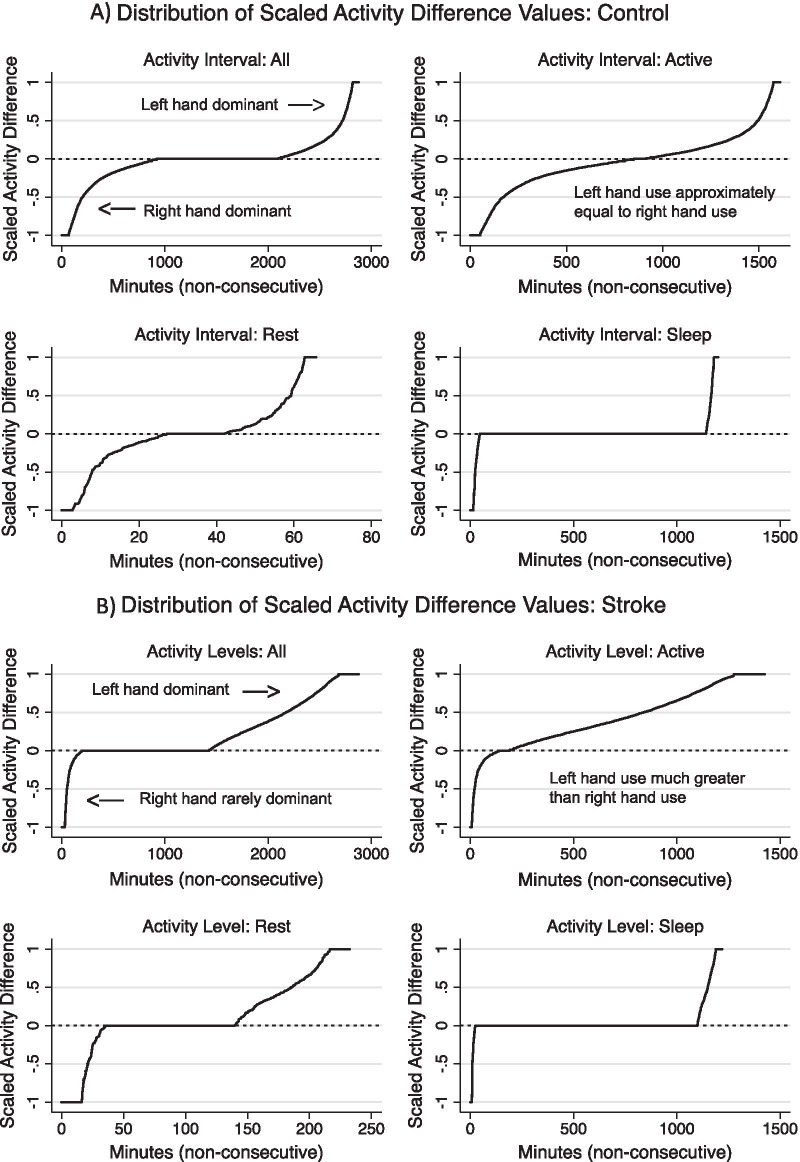
Distribution of Scaled activity difference. All SAD values ranged from − 1 to 1; values of 1 indicate left arm use only, values of − 1 indicate right arm use only, values of 0 indicate equal left and right arm use, values between 0 and 1 indicate left arm use is dominant, and values between 0 and − 1 indicate right arm use is dominant. **A** Typically-developing participant: Note the high degree of rotational symmetry in all graphs, indicating nearly equal use of left and right arms. **B** Participant with stroke: Movement is markedly asymmetric with a low degree of rotational symmetry, indicating that left arm use is much greater than right arm use

A moment-by-moment asymmetry measure, which we label the Scaled activity difference (SAD), was calculated:

$$Scaled\,activity\,difference \left(SAD\right)= \frac{{L}_{it}- {R}_{it}}{{L}_{it}+{R}_{it}}$$

where *L*_*it*_ and *R*_*it*_ are the AC for the left and right hands in epoch of time *t* for individual *i.* SAD was zero when there was no activity in either hand. Thus, SAD values ranged from − 1 to 1 with − 1 and 1 indicating only right and left arm movement, respectively, and values of 0 indicating equal left and right arm use. A SAD value was generated for each 15-s epoch for each participant. The SAD is similar to the “Asymmetry Index” used by Beani et al. and elsewhere, but assesses asymmetry at a given epoch, rather than as an average value [23]. For each individual, the SAD statistics were graphed to create a visual representation of the SAD score distribution for that participant, after being ranked from smallest to largest (Fig. 1).

We also constructed a novel summary statistic called the Actigraphic Movement Asymmetry Index (AMAI) as:

$$AMAI = 1- \left|{\left(mean SAD\right)}_{ijk}\right|$$

for interval *j* and level *k* for individual *i.* The AMAI generated a single value to represent bilateral movement asymmetry in each participant for each interval *j* and level *k*. Values range from 0 to 1 with closer to 1 indicating greater symmetry, a value of 1 indicating perfect symmetry, and a value of 0 indicating completely unilateral movement (fully asymmetric). A key feature of the AMAI is that it gives equal weight to all epochs regardless of intensity of upper limb activity. This accords value to both large and small movements, which are each important in everyday life. The AMAI was the primary outcome.

### Standard motor outcomes

Standardized clinical motor outcomes, the AHA, MA and BB, were obtained by experienced pediatric occupational therapists within the context of a clinical trial [9, 10]. Measuring therapists were blinded to all patient details including stroke type, size and location at the time of assessment. Only the summary score for each test was used in the current analysis. Most BB scores were obtained during the trial measures; seven additional participants who were otherwise eligible but lacked recent AHA and MA scores received BB assessments from a trained research assistant. We also calculated the “Block Ratio” statistic, as described by Raglio [31] to provide a single value for BB scores and enable comparison with the AMAI:

$$Block\,Ratio= \frac{\#\,of\,blocks \,moved \,in \,1\, min\, by\, affected\, hand}{\# \,of\, blocks\, moved \,in \,1 \,min\, by \,unaffected \,hand}$$

### Statistical analysis

Associations were tested using Pearson correlation if data was normally distributed (Shapiro–Wilk test) and Spearman correlation if it was not. First, we examined correlations between different AMAI (actigraphy) intervals and levels. Second, we examined correlations between standard outcomes (AHA, MA, BB). Third, we tested for correlations between AMAI and the AHA, MA, and BB for all combinations of activity intervals (active, rest, sleep, all) and levels (very low, low, moderate, high, very high, all) for stroke participants only. No adjustments were made for participants without AHA or MA scores. Scatterplots provided visual representations of selected relationships between variables.

Box plots of the AMAI compared differences between groups across activity intervals. Welch’s *t*-test (unequal variances *t*-test) and Mann–Whitney U tests were applied depending on normality to compare groups. The tests were performed to systematically compare AMAI between participants with stroke and typically-developing participants. As our primary outcome (AMAI) has not been previously described, a formal power calculation was not possible. However, based on similar approaches in the literature, a minimum sample of 25 stroke participants was estimated. Analysis was performed using Stata (version 14.2).

## Results

### Populations

53 individuals participated. Group characteristics, demographics and motor outcomes are summarized in Table 1. 30 stroke participants had a mean age of 11 years 2 months (SD 3 years 10 months; range 3 years 10 months–17 years 10 months; 13 female, 17 male). 23 typically-developing participants had a mean age of 11 years 1 month (SD 4 years 5 months; range 1 year 4 months–17 years 11 months; 8 female, 15 male). Groups did not differ significantly in age (*p* = 0.97) or sex (*p* = 0.53). The AMAI was calculated for all participants. All three motor outcomes were obtained for 21 stroke participants while 7 had BB scores only. There were no adverse events and no drop-outs, and all stroke and control participants fully or partially filled out the diary.

**Table 1:** Participant recruitment and characteristics

	Participants with stroke	Typically-developing participants
Eligible (*n*)	55	109
Approached (*n*)	44	37
Enrolled (*n*)	30	23
Actigraphy data (*n*)	30	23
Full set of AHA, MA and BB Scores (*n*)	21	N/A
BB scores only (No AHA or MA) (*n*)	7	N/A
Participants with BB scores (*n*)	28	N/A
Diary partially/fully completed (*n*)	30	23
Sex (*n*)	Male (17), Female (13)	Male (15), Female (8)
Age range	3 years 10 months–17 years 10 months	1 year 4 months–17 years 11 months
Median age	11 years 6 months	12 years 2 months
Mean age (SD)	11 years 2 months (3 years 10 months)	11 years 1 month (4 years 5 months)

Actigraphy data were collected from all stroke and control participants. Standard test scores were only available for the stroke cohort, and BB scores were either collected or available for 28/30 participants. 21 of those participants also had AHA and MA scores. The control cohort had a slightly wider age range than the stroke cohort, although the median and mean ages were similar. There were more males than females in both cohorts

### Actigraphy data

The majority of epochs (57.7% stroke; 60.9% control) were distributed in active intervals between low and moderate activity levels. A large proportion of epochs (38.6% stroke; 36.3% control) occurred in sleep intervals with very low activity levels. Within sleep, epochs of low to moderate activity levels were also present, but decreased in frequency as activity level increased. The overall distribution of epochs was comparable across both groups (Additional file 1: Table B). Participants wore the watches for an average of 96.7% of the time, with excluded time allowing for bathing or other activities involving water.

#### Scaled activity difference (SAD)

Scaled activity difference plots visually represent asymmetry of upper extremity movement across participants (Fig. 1). Patterns of asymmetry can be seen in SAD curves across all intervals. Movement curves among participants with stroke were characterized by rotational asymmetry. Comparison of SAD plots between participants with stroke and typically-developing participants visually represented the degree and nature of movement asymmetry among participants with stroke.

#### Actigraphic movement asymmetry index (AMAI)

**Fig. 2:**
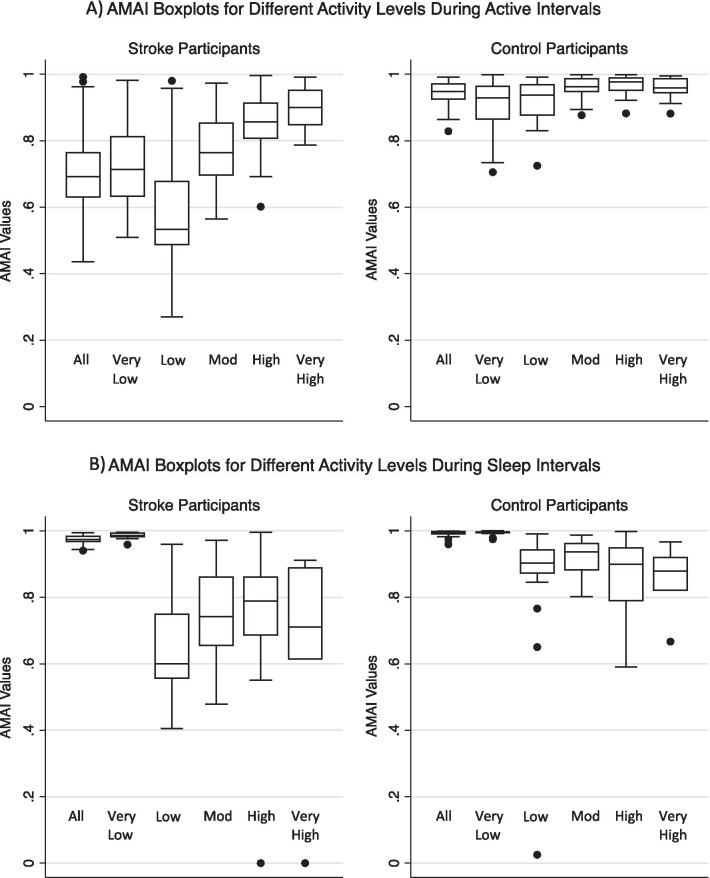
Distribution of AMAI scores during active (**A**) and sleep (**B**) intervals. Scores range from 0 to 1; values approaching 1 indicate greater symmetry, values of 1 indicate perfect symmetry, and values of 0 indicate completely unilateral movement (perfectly asymmetric). Differences between stroke and control participants were statistically significant (α = 0.05) for each activity level (see Additional file 1: Table A for relevant statistical test results)

**Fig. 3:**
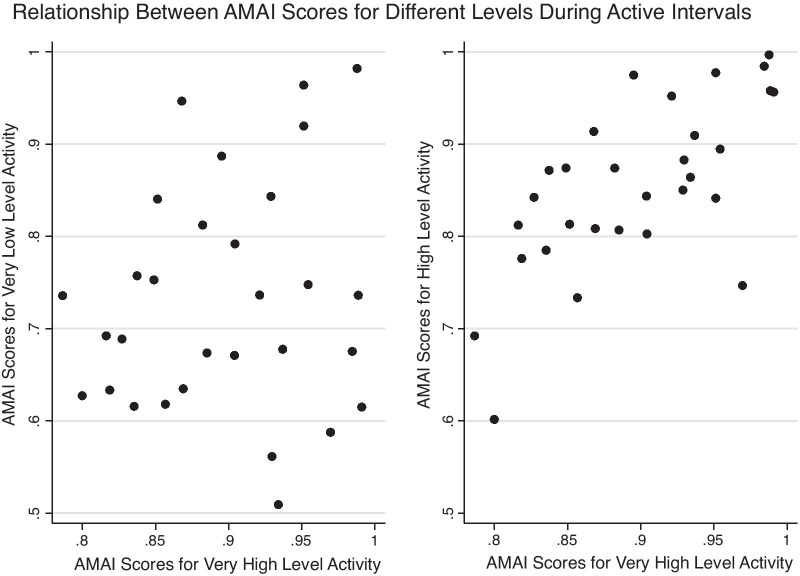
Selected relationships for AMAI scores in active intervals. The scatter plot on the left represents the relationship between the AMAI “very low” and “very high” levels; the scatter plot on the right represents the relationship between the AMAI “high” and “very high” levels. Only the graph on the right shows a strong relationship, consistent with Additional file 1: Table C

AMAI scores spanned most of the possible range (from 0.03 to 0.99 out of 1) indicating that participants ranged from very asymmetric to highly symmetric. AMAI scores were widely distributed for participants with stroke but not for typically-developing participants, whose scores clustered near 1. Comparison of mean AMAI scores for stroke and typically-developing groups revealed statistically significant differences; the typically-developing group had higher symmetry across activity levels (Fig. 2, Additional file 1: Table A). The lone exception was high and very high levels during rest/sleep; however, these sections contained very limited data, typically < 0.1% of all data collected. AMAI scores correlated across activity levels with each other. Correlations were strongest between adjacent  activity levels while strength of correlation decreased the further the activity levels diverged (Fig. 3, Additional file 1: Table C).

#### AMAI versus standard motor outcomes

Modest correlations between standard tests and AMAI values were consistently observed. All motor outcomes correlated with the AMAI, but the highest associations were observed with the Block Ratio. Associations were strongest during sleep and rest intervals, and weaker but still significant during active intervals. Within sleep intervals, the strongest correlations were present in low and moderate activity levels (e.g. sleep, low activity, *r* = 0.68, *p* < 0.01). Correlations were weaker for active intervals with very low or low activity levels (e.g. *r* = 0.42, *p* = 0.03) and not significant for very high activity levels (e.g. *r* = 0.21, *p* = 0.28) (Table 2). Strong correlations during sleep intervals remained after stratification by age. Low or moderate activity levels were most consistently correlated with the three motor outcomes, which also correlated strongly with each other. The strongest correlations were observed between the Block Ratio and the AHA and MA (*r* = 0.93 and 0.87 respectively). Correlation between the AHA and MA was also significant (*r* = 0.82).

**Table 2:** Master correlation table

Interval	Level	BB		MA		AHA	
		r	p	r	p	r	p
Active	Very low	**0.42**	0.026	0.39	0.081	0.34^†^	0.132
	Low	**0.49**	0.008	0.4	0.07	0.36^†^	0.112
	Moderate	0.35	0.068	0.33	0.143	0.32^†^	0.152
	High	0.23	0.243	0.12	0.606	0.21^†^	0.36
	Very high	0.21	0.284	0.06	0.792	0.08^†^	0.741
	All	**0.42**	0.027	0.39	0.076	0.40^†^	0.071
Sleep	Very low	0.17^†^	0.45	0.10^†^	0.68	0.12^†^	0.607
	Low	**0.68**	< 0.001	**0.57**	0.007	**0.60**^**†**^	0.004
	Moderate	**0.56**	0.003	**0.48**	0.027	**0.50**^**†**^	0.021
	High	0.49^†^	0.086	0.42^†^	0.149	0.44^†^	0.135
	Very high	0.12	0.883	N/A	N/A	N/A	N/A
	All	**0.43**	0.026	0.41	0.066	**0.48**^**†**^	0.027
Rest	Very low	0.27^†^	0.234	0.33^†^	0.15	0.30^†^	0.191
	Low	**0.4**	0.04	0.2	0.381	0.31^†^	0.168
	Moderate	**0.57**	0.002	**0.69**	0.001	**0.74**^**†**^	< 0.001
	High	0.26	0.215	0.28	0.259	0.20^†^	0.434
	Very high	**0.67**^**†**^	0.05	**0.67**^**†**^	0.05	0.40^†^	0.284
	All	0.35	0.071	0.35	0.118	**0.50**^**†**^	0.02
All	Very low	0.20^†^	0.375	0.24^†^	0.302	0.19^†^	0.419
	Low	0.42^†^	0.056	0.39^†^	0.081	0.37^†^	0.101
	Moderate	0.36	0.058	0.35	0.12	0.32^†^	0.151
	High	0.24	0.216	0.14	0.546	0.24^†^	0.293
	Very high	0.21	0.273	0.07	0.769	0.10^†^	0.657
	All	**0.46**	0.014	0.35	0.117	0.33^†^	0.15

Pearson/Spearman Correlations between clinical motor outcomes and the AMAI. Correlations that were significant at the α = 0.05 level are shown in bold text. Levels were defined for each 15-s epoch by the sum of the Activity Counts for both hands: [a] very low (total of 0–30), [b] low (31–160), [c] moderate (161–524), [d] high (525–812), [e] very high (813+), and [f] all

^†^Denotes Spearman’s rho

## Discussion

Our prospective cohort study suggests that bilateral wrist-worn actigraphs can measure asymmetry of upper limb movements in hemiparetic children during everyday life activities. Both the SAD graph and AMAI quantify characteristics of upper limb movements across activity levels. Correlations of varying strength between actigraphic data and standard assessments (BB, MA, AHA) suggests that standard assessments may not fully reflect real-life movement asymmetry. Actigraphy appears to be a practical way of capturing upper extremity movement both within clinical trials and normal life.

Bilateral actigraphy may offer some advantages for measuring upper extremity function in hemiparetic children. Actigraphy is not contaminated by learning and practice effects that may occur with repeated administration of standard measures. Actigraphy appears to represent a wide range of movement with continuous, objective measurement of movement asymmetry. Application before and after therapeutic interventions or longitudinally during development may be particularly useful. Varying strength of correlation between standard functional tests may indicate that standard tests do not fully reflect movement in everyday life. For example, correlations were strongest during sleep, a finding which may deserve further study. It is important to note that actigraphy metrics reflect different domains of functioning within the ICF. Specifically, actigraphy measures body functions (rather than activity) in real-world environments (rather than institutional environments). Thus actigraphy may be an important complement to standard tests. Whether actigraphy reflects what matters to individual children and families requires further study.

Consistent with existing literature in the field, our data are consistent with the feasibility of bilateral actigraphy in children: (1) no adverse effects were reported, (2) all participants were able wear the watches for many consecutive hours, and (3) all participants were able to fill out the diary tracking actiwatch use. Children, particularly those of school age, generally subjectively reported enjoying wearing the devices. Participant recruitment and enrolment were uncomplicated, and teaching families and arranging unit collection and return was not onerous.

Our study developed practical metrics to assess motor asymmetry during a wide variety of movement levels and types using actigraphy. The graphical display of SAD statistics appears to be a valuable way to transform raw actigraphy data into an easily interpreted visual representation of a wide range of asymmetries in bilateral upper extremity movement. Shifts in SAD curves may represent an informative new tool to examine the effects of interventions designed to increase real-world upper extremity use. The AMAI offers a single representative summary statistic per participant, enabling examination of movement within individuals across activity levels and between groups. The wide range of scores observed appears to reflect the expected spectrum of physical disability. Application across populations and within interventional trials will be required to determine the utility of the SAD graphs and AMAI. Additionally, another potential avenue of use may include motor function assessment or even early diagnosis of cerebral palsy in very young children.

Important limitations are acknowledged. Actigraphy outcomes were only compared with available standard tests, which themselves may be imperfect. The fact that actigraphy data correlated with, but were different from, these established measures does not mean one is necessarily better than the other. As discussed earlier, these tools likely reflect different aspects of upper extremity function. Other limitations include our modest sample size which may have limited our ability to fully define the utility of actigraphic measurements. For some participants, there was a delay of several months between the collection of our standard test scores and actigraphy data which may have affected the correlations reported. Our actiwatches were also not designed to capture brief, smaller hand movements, which occur over a few seconds and were beyond the temporal resolution of our methods [13, 16]. In addition, although actigraphy may capture movement asymmetry, further validation is needed to verify that activity counts fully reflect functional limb use: actigraphy may be biased by impaired movement patterns, resulting in an elevated movement count not attributable to functional use.

## Conclusions

Overall, three main findings emerged from this research. First, actigraphy appears to be able to measure daily-life motor asymmetry in both typically-developing and hemiparetic children. Second, using the SAD graphs and AMAI statistics, actigraphy can assess the range of everyday bilateral upper-extremity motor asymmetries. Finally, the diversity in everyday movement of children with hemiparesis captured by actigraphy suggests that the standard tests may not fully represent movement asymmetries.

The translational value of detecting change in real-world asymmetry of upper limb movement in hemiparetic children may be game-changing. Modern models of pediatric rehabilitation have moved towards intensive treatment interventions over short time periods, often attempting to deliver high doses of therapy over weeks. While such approaches can yield measurable changes in function according to standard measures, effect sizes are often modest with only a portion of subjects “responding”. However, more encouraging is evidence that small changes produced in the short-term grow over subsequent months and years. Our recent trial of constraint therapy and brain stimulation in hemiparetic children observed this phenomenon with sustained or increased gains at 6 months [9]. Even in the developed brain, the effects of 2 weeks of constraint therapy in adult stroke hemiparesis have been shown to increase at 2 years follow-up [32]. These results suggest that small increases in the use of a hemiparetic limb resulting from an acute intervention may amplify over time to realize more significant long-term functional benefits. If true, such effects are almost certainly more important in the developing brains of young children. Bilateral actigraphy brings new promise to better measure and understand such developmental effects of neurorehabilitation.

In addition to the replication of our findings, important future directions include the use of bilateral actigraphy in prospective, controlled clinical trials of pediatric hemiparesis. We are employing this approach in a current multi-center clinical trial of non-invasive brain stimulation combined with intensive therapy in hemiparetic children with perinatal stroke (NCT03216837). Such efforts to validate actigraphy hold promise in the development of real-life measures of rehabilitation interventions.

## Supplementary Information

**Additional file 1: Table A.** Comparing Mean AMAI Scores for stroke and control cohorts. **Table B.** Distribution of Epochs. **Table C.** Pearson correlations between different AMAI activity levels in active intervals for typically-developing participants.

## Data Availability

The datasets used and/or analysed for this study are available from the corresponding author upon reasonable request.

## Abbreviations

AHA: Assisting Hand Assessment
AMAI: Actigraphic Movement Asymmetry Index
BB: Box and Blocks Test
CP: Cerebral palsy
HCP: Hemiparetic cerebral palsy
HICCUP: Healthy Infants and Children Clinical Research Program
ICF: International Classification of Functioning, Disability, and Health
MA: Melbourne assessment
SAD: Scaled activity difference
