# Supplementary material for: Bilateral actigraphic quantification of upper extremity movement in hemiparetic children with perinatal stroke: a case control study
Source: J Neuroeng Rehabil. 2021 Dec 16;18:172. doi: 10.1186/s12984-021-00962-9 (PMC8680110; doi:10.1186/s12984-021-00962-9)
Supplement: Supplementary file 1 — Additional file 1: Table A. Comparing Mean AMAI Scores for stroke and control cohorts. Table B. Distribution of Epochs. Table C. Pearson correlations between different AMAI activity levels in active intervals for typically-developing participants. [file 12984_2021_962_MOESM1_ESM.docx]

ADDITIONAL FILES:

Table A: Comparing Mean AMAI Scores for Stroke and Control Cohorts.

| **Interval** | **Level** | **Stroke Mean** (**SD)** | **Control Mean**±**SD** | **p-value from t- or u-test** | **Difference in Means (95% CI)** |
| --- | --- | --- | --- | --- | --- |
| Active | All | 0.713 (0.13) | 0.941 (0.04) | < 0.001 | 0.228 (0.18 – 0.29) |
|  | Very Low | 0.731 (0.12) | 0.902 (0.08) | < 0.001 | 0.171 (0.11 – 0.23) |
|  | Low | 0.593 (0.17) | 0.918 (0.06) | < 0.001 | 0.325 (0.26 – 0.39) |
|  | Moderate | 0.775 (0.12) | 0.960 (0.03) | < 0.001 | 0.185 (0.14 – 0.23) |
|  | High | 0.855 (0.09) | 0.969 (0.03) | < 0.001 | 0.114 (0.08 – 0.15) |
|  | Very High | 0.897 (0.06) | 0.958 (0.03) | < 0.001† | 0.061 (0.04 – 0.09) |
| Rest | All | 0.826 (0.10) | 0.949 (0.05) | < 0.001 | 0.122 (0.08 – 0.16) |
|  | Very Low | 0.886 (0.10) | 0.952 (0.05) | 0.001 | 0.066 (0.02 – 0.11) |
|  | Low | 0.648 (0.16) | 0.904 (0.08) | < 0.001 | 0.256 (0.18 – 0.33) |
|  | Moderate | 0.768 (0.13) | 0.902 (0.10) | < 0.001 | 0.134 (0.07 – 0.20) |
|  | High | 0.736 (0.13) | 0.859 (0.17) | < 0.001 | 0.123 (0.03 – 0.22) |
|  | Very High | 0.765 (0.24) | 0.857 (0.12) | 0.198† | 0.092 (-0.05 – 0.23) |
| Sleep | All | 0.973 (0.01) | 0.992 (0.01) | < 0.001 | 0.019 (0.01 – 0.02) |
|  | Very Low | 0.986 (0.01) | 0.995 (0.01) | < 0.001 | 0.008 (0.00 – 0.01) |
|  | Low | 0.644 (0.14) | 0.858 (0.20) | < 0.001 | 0.214 (0.12 – 0.31) |
|  | Moderate | 0.754 (0.14) | 0.926 (0.05) | < 0.001 | 0.172 (0.12 – 0.23) |
|  | High | 0.743 (0.21) | 0.863 (0.12) | 0.044† | 0.120 (0.00 – 0.24) |
|  | Very High | 0.625 (0.37) | 0.856 (0.11) | 0.242† | 0.230 (-0.22 – 0.68) |
| All | All | 0.818 (0.08) | 0.963 (0.02) | < 0.001 | 0.154 (0.11 – 0.18) |
|  | Very Low | 0.910 (0.06) | 0.976 (0.02) | < 0.001 | 0.066 (0.04 – 0.09) |
|  | Low | 0.600 (0.17) | 0.923 (0.06) | < 0.001 | 0.323 (0.25 – 0.39) |
|  | Moderate | 0.754 (0.14) | 0.926 (0.05) | < 0.001 | 0.172 (0.12 – 0.23) |
|  | High | 0.743 (0.21) | 0.863 (0.12) | < 0.001 | 0.120 (0.00 – 0.24) |
|  | Very High | 0.898 (0.06) | 0.958 (0.03) | < 0.001† | 0.061 (0.04 – 0.09) |

In almost all levels and intervals, AMAI scores for the stroke and control cohorts were statistically significantly different, based on results for Welch’s t-test and Mann-Whitney u-tests. † denotes p-value from Welch’s t-test.

Table B: Distribution of Epochs.

|  | Stroke | | | Control | | |
| --- | --- | --- | --- | --- | --- | --- |
|  | **Active** | **Rest** | **Sleep** | **Active** | **Rest** | **Sleep** |

| **Very Low** | 13.24% | 2.21% | 37.04% | 10.10% | 1.72% | 34.15% |
| --- | --- | --- | --- | --- | --- | --- |
| **Low** | 19.90% | 0.89% | 1.18% | 19.54% | 0.58% | 1.51% |
| **Moderate** | 18.80% | 0.50% | 0.40% | 22.86% | 0.41% | 0.61% |
| **High** | 3.75% | 0.06% | 0.02% | 4.91% | 0.04% | 0.03% |
| **Very High** | 2.00% | 0.02% | 0.00% | 3.51% | 0.01% | 0.01% |

The average proportions of 15-second epochs for the stroke and control cohorts are shown. The greatest proportions of epochs occurred during the active intervals of low and moderate levels, and the sleep intervals of very low levels. Note that 1% is approximately 112 epochs.

Table C: Pearson correlations between different AMAI activity levels in active intervals for typically-developing participants.

|  | **Very Low** | **Low** | **Moderate** | **High** | **Very High** |
| --- | --- | --- | --- | --- | --- |
| **Very Low** |  |  |  |  |  |
| **Low** | *r*=0.70  *p* < 0.001 |  |  |  |  |
| **Moderate** | *r*=0.62  *p* < 0.001 | *r*=0.91  p < 0.001 |  |  |  |
| **High** | *r*=0.43  *p* = 0.019 | *r*=0.70  p < 0.001 | *r*=0.705  p < 0.001 |  |  |
| **Very High** | *r*=0.15  p = 0.444 | *r*=0.355  p = 0.054 | *r*=0.47  p = 0.009 | *r*=0.68  p < 0.001 |  |

Strongest correlations were present between the levels that were closer together or more similar, such as low and very low, or moderate and low; weaker correlations were present between the levels that were father apart or more different, such as very low and very high.
